# Vectorized Delivery of Alpha-GalactosylCeramide and Tumor Antigen on Filamentous Bacteriophage fd Induces Protective Immunity by Enhancing Tumor-Specific T Cell Response

**DOI:** 10.3389/fimmu.2018.01496

**Published:** 2018-06-28

**Authors:** Rossella Sartorius, Luciana D’Apice, Pasquale Barba, Deborah Cipria, Laura Grauso, Adele Cutignano, Piergiuseppe De Berardinis

**Affiliations:** ^1^Institute of Protein Biochemistry, CNR, Naples, Italy; ^2^Institute of Genetics and Biophysics “A. Buzzati Traverso”, Naples, Italy; ^3^Institute of Biomolecular Chemistry (ICB), CNR, Pozzuoli, Italy

**Keywords:** vectorized alpha-GalactosylCeramide, filamentous bacteriophage, invariant Natural Killer T cells, anti-tumor immunity, CD8^+^ T Cells

## Abstract

We have exploited the properties of filamentous bacteriophage *fd* to deliver immunologically active lipids together with antigenic peptides. Filamentous bacteriophages resemble for size, capability to be permeable to blood vessels, and high density antigen expression, a nature-made nanoparticle. In addition, their major coat protein pVIII, which is arranged to form a tubular shield surrounding the phage genome, has a high content of hydrophobic residues promoting lipid association. We conjugated bacteriophages to alpha-GalactosylCeramide (α-GalCer), a lipid antigen-stimulating invariant natural killer T (iNKT) cells and capable of inducing their anti-tumoral activities. We found that bacteriophage fd/α-GalCer conjugates could repeatedly stimulate iNKT cells *in vitro* and *in vivo*, without inducing iNKT anergy. Moreover, co-delivery of α-GalCer and a MHC class I restricted tumor-associated antigenic determinant to antigen-presenting cells *via* bacteriophages strongly boosted adaptive CD8^+^ T cell response and efficiently delayed tumor progression. Co-delivery of a tumor antigen and iNKT-stimulatory lipid on the surface of filamentous bacteriophages is a novel approach to potentiate adaptive anti-cancer immune responses, overcoming the current limitations in the use of free α-GalCer and may represent an attractive alternative to existing delivery methods, opening the path to a potential translational usage of this safe, inexpensive, and versatile tool.

## Introduction

Invariant natural killer T (iNKT) cells represent a unique subpopulation of T lymphocytes with both innate-like and adaptive functions mainly found in spleen, liver, and bone marrow. iNKT cells express NK lineage receptors and a semi-invariant T cell receptor (TCR) composed of Vα14–Jα18 chain in mice and Vα24–Jα18 chain in humans, paired with β chains encoded by a limited number of Vβ genes (1).

Invariant natural killer T cells recognizes (glyco)lipid antigens (Ag) presented by the CD1d molecule expressed by several types of antigen-presenting cells (APCs), including dendritic cells (DCs) (2).

The potential anti-tumoral function of iNKT cells was first discovered with the identification of alpha-GalactosylCeramide (α-GalCer), a synthetic derivative of agelasphin, a glycolipid originally isolated from the marine sponge *Agelas mauritianus*, as a strong stimulatory ligand during screening of anti-tumor compounds from natural sources (3–5).

In response to α-GalCer, iNKT cells rapidly secrete large quantities of cytokines, including IFN-γ, IL-2, IL-4, IL-6, IL-10, IL-12, IL-13, IL-17, IL-21, IL-22, TGF-β, and TNF-α, that in turn activate a variety of other cell types, including NK cells, DCs, B, and T cells (6–9). Through this activation cascade, α-GalCer showed to exert potent anti-tumor and adjuvant activities *in vivo* in mouse models (5, 10, 11), rendering it a powerful candidate for adjuvant therapy in cancer.

Based on these pioneering reports, attempts have been made to exploit the anti-tumor property of α-GalCer. Although the drug was well tolerated, no or moderate clinical responses were observed among the patients repeatedly injected intravenously with α-GalCer (12). Studies performed in a mouse model demonstrated that α-GalCer induced a long-term anergy of iNKT cells, thus preventing proliferation and cytokine release upon a recall stimulation (13).

Even though the mechanism underlying iNKT cell anergy mediated by α-GalCer is still unknown, it is common knowledge that co-stimulatory signals and cytokines provided by antigen-presenting DCs are considered crucial for avoiding anergy (14).

Indeed, mouse studies demonstrated that the injection of α-GalCer-pulsed DCs induced a sustained cytokines production when compared with administration of free α-GalCer, suggesting that the type of α-GalCer administration is critical for iNKT cell stimulation (15).

A way to optimize iNKT cell responses may lie in actively directing α-GalCer to the appropriate APC using suitable delivery systems (16).

In previous studies, we found that the filamentous bacteriophage is an efficient antigen-delivery system because it is internalized by DCs and activates innate and adaptive immune responses in the absence of classical adjuvants (17–19).

The filamentous bacteriophage can be considered as a nature-made nanocarrier according to its nano-dimensions (5 nm in diameter and 1,000 nm in length), its capability to cross blood vessels and for the capacity of expressing very large amounts of recombinant protein antigen. Its major coat protein pVIII is present in 2,700 copies on the phage coat and is arranged to form a tubular shield surrounding the phage genome. The pVIII protein is composed of three specific domains: a hydrophobic core, an acidic N-terminal domain, and a basic C-terminal domain (20). Due to the high content of hydrophobic residues, the pVIII shows strong binding of lipids, a feature that we exploited by promoting association of the bacteriophage with α-GalCer. Here, we show that α-GalCer conjugated with bacteriophages could repeatedly stimulate iNKT cells *in vitro* and *in vivo*, without inducing iNKT cell anergy. Moreover, therapeutic vaccination with phages co-displaying α-GalCer and a tumor antigen delayed tumor progression in mice, showing improved adjuvanticity of the phage particles and leading to enhanced vaccine efficacy. These results suggest a novel approach to potentiate the efficacy of the bacteriophage delivery system as anti-tumor vaccine.

## Materials and Methods

### Cells and Reagents

CD1d-restricted Vα14i NKT hybridoma FF13 (21) cells were a kind gift of Dr. De Libero (Department of Biomedicine, University of Basel and University Hospital Basel, Basel, Switzerland) and were cultured in RPMI 1640 (Lonza) medium supplemented with 100 U/ml penicillin, 100 µg/ml streptomycin, and 10% fetal calf serum (FCS) (all from GIBCO, Milan, Italy).

OTI hybridoma cell line, recognizing the OVA_257–264_ SIINFEKL determinant, was produced by infection of 54ζ17 hybridoma T cells (22) with a retrovirus encoding the OTI TCR V alpha 2 and V beta 5 chains. Recombinant retroviral particles were produced in HEK293T cells (ATCC CRL 1573) by transfection with pMXOTI, pEcotropic, and pVSVg plasmids. The virus-containing supernatant was collected 48 h after transfection. 5 × 10^5^ 54ζ17 cells were infected with 10 ml of viral supernatant and TCR expression was verified after 5 days by APC-anti-CD3ε (145-2C11, Biolegend) staining and FACS analysis. Positive cells were then sorted using a FACS ARIA (Becton Dickinson, Fullerton, CA, USA) and amplified in DMEM supplemented with 10% FCS, 25 U/ml penicillin G, 25 µg/ml streptomycin, and 0.05 µM β-mercaptoethanol.

B16-OVA melanoma cells (H2Kb), stably expressing chicken ovalbumin, were a kind gift of Dr. Dellabona (San Raffaele Scientific Institute, Milan, Italy). Cells were cultured in RPMI 1640 medium supplemented with 100 U/ml penicillin, 100 µg/ml streptomycin, 10% FCS, and 100 µg/ml Hygromicin (Sigma-Aldrich).

The synthetic peptide OVA_257–264_ (SIINFEKL) was purchased from Primm (Naples, Italy).

Synthetic α-GalCer (KRN7000) (*2S,3S,4R*)-1-*O*-(α-D-galactosyl)-*N*-hexacosanoyl-2-amino-1,3,4-octadecanetriol (BML-SL232-1000) was purchased from Vinci Biochem.

Internal standard (IS) for mass spectrometric analysis D-Galactosyl-β-1,1’-*N*-Palmitoyl-D-erythro sphingosine was purchased from Avanti Polar Lipids.

MeOH and water for liquid chromatography–mass spectrometric analysis were purchased from Merck and were LC–MS grade.

### Mice

Six- to eight-week-old female C57BL/6 were purchased from Charles River (Lecco, Italy) and housed in IGB “A. Buzzati-Traverso” Animal House Facility under standard pathogen-free conditions abiding institutional guidelines.

### Purification of Bacteriophages Particles and Conjugation to α-GalCer

Wild type (fdWT) and hybrid fdOVA (expressing the recombinant OVA_257–264_-pVIII proteins) filamentous bacteriophages were purified from the supernatant of transformed *Escherichia coli* TG1recO cells. Bacteria were grown in TY2X medium for 16 h and the bacteriophage virions were harvested from *E. coli* supernatant, Poly(ethylene glycol) 6000 (Sigma-Aldrich) precipitated, purified by ultracentrifugation (24,500 *g*) on cesium chloride (Sigma-Aldrich) gradient, and dialyzed against phosphate buffered saline (PBS) 1×. Elimination of lipopolysaccharides (LPS) from phage particles was performed using Triton X-114 (Sigma-Aldrich). Briefly, Triton X-114 was mixed to the phage preparations to a final concentration of 1% by vigorous vortexing. The mixture was incubated at 4°C for 5 min, then incubated for 5 min at 50°C, and centrifuged (20,000 *g*, 10 min) at 25°C. The upper aqueous phase containing the virions was carefully removed and subjected to Triton X-114 phase separation for more cycles. The resulting aqueous phase containing virions was subjected to cesium chloride gradient centrifugation, dialyzed against PBS 1×, and assayed for LPS contamination using the Limulus Amebocyte Lysate assay (Limulus Amebocyte Lysate QCL-1000 chromogenic modification, Lonza), according to the manufacturer’s instructions.

The expression of the recombinant OVA_257–264_-pVIII proteins was induced adding 0.1 mM isopropyl-beta-D-thiogalactopyranoside (Sigma-Aldrich) to the bacteria growing in TY2X medium.

The number of copies of pVIII displaying the OVA_257–264_ peptide was estimated by N-terminal sequence analysis of the purified virions and resulted in 15–20% for each phage preparation.

Bacteriophages in PBS 1× pH 8 and KRN7000 in dimethyl sulfoxide (Sigma-Aldrich) were combined at a 10:1 ratio (μg phages: μg α-GalCer) and stirred at 4°C overnight. Virions were subjected to cesium chloride gradient ultracentrifugation (24,500 *g*), dialyzed against PBS 1× and the concentration of bacteriophages was determined using spectrophotometer. The presence of α-GalCer in the phage preparations was determined by the *in vitro* biological assay and its conjugated amount determined by mass spectrometric analysis, as described below.

### Release of α-GalCer From the Conjugated Bacteriophage by Solvent Extraction

A small aliquot (50 µl) of a PBS solution containing the bacteriophage conjugated to α-GalCer at a concentration of 1.5 mg/ml was diluted 1:10 to a final volume of 500 µl with ultrapure water; 200 ng of IS in 20 µl of methanol (MeOH) were added and the suspension was extracted with 2 ml of MeOH/CHCl_3_ (1:1) by sonication. After centrifugation, the organic phase was removed and the aqueous layer was re-extracted with MeOH/CHCl_3_. The combined organic phases were dried under nitrogen, reconstituted in 1 ml of MeOH, and subjected to LC–MS analysis.

### Quantitative LC–MS/MS Analysis of α-GalCer

A quantitative method was developed on a UPLC system (Acquity, Waters) coupled to a triple quadrupole mass spectrometer (API 3200, SCIEX). The chromatographic analysis was performed on an Acquity BEH Phenyl column (Waters, 100 × 2.1 mm, 1.7 µm), eluted with a short gradient program from 95:5 MeOH/H_2_O to 100% MeOH in 1 min followed by an isocratic elution at 100% MeOH for 4 min. Flow rate was set at 0.4 ml/min and column temperature at 40°C. α-GalCer eluted at a Rt of 1.59 min, IS at 1.1 min.

A calibration curve was prepared by using five calibration points of α-GalCer standard (STD) (62.5, 125, 250, 500, and 1,000 ng/ml) spiked with 200 ng/ml IS and plotted as area ratio of STD/IS response vs concentration. Two MRM transitions were monitored for both STD and IS for quantitative purposes and to confirm analytical identification. The most intense transitions for each compound (i.e., *m/z* 856.7 > 178.9 for STD and *m/z* 698.5 > 89.2 for IS) were used as analytical responses.

### *In Vitro* α-GalCer Presentation on CD1d and Stimulation of iNKT and OTI Hybridoma Cells

Mouse bone marrow-derived dendritic cells (BMDCs) were generated from C57BL/6 mice according to Ref. (23). At 7 day of culture, BMDCs were incubated in RPMI medium supplemented with 10% FCS, 5 µM 2-ME, 1 mM glutamine, and 1 mM sodium pyruvate for 2 h with different concentrations of free α-GalCer, fdWT bacteriophages, or fd/α-GalCer bacteriophages. The experiment OTI hybridoma cell experiment was performed by incubating BMDCs with different concentration of fdOVA, fdOVA/α-GalCer, or OVA_257–264_ synthetic peptide. After the incubation, cells were washed and stained with PE-conjugated anti mouse α-GalCer:CD1d complex (L363, Biolegend) or co-cultured (50,000/well) with the mouse Vα14 iNKT hybridoma FF13 or OTI hybridoma (50,000/well) for 40 h.

PE-conjugated anti-mouse α-GalCer:CD1d complex (L363, Biolegend) antibody was used to stain DCs and fluorescence of stained cells was analyzed by FACSCanto II flow-cytometer and DIVA (Data-Interpolating Variational Analysis) software (Becton Dickinson). The IL-2 released into cell co-culture supernatants was measured by ELISA. Supernatants (0.1 ml/well) were assayed in duplicate using mouse IL-2 ELISA MAX™ Standard (Biolegend), according to the manufacturer’s instructions.

### Measurement of *In Vivo* and *In Vitro* Recall Response to α-GalCer

Mice were injected intravenously (i.v.) or intratumorally (i.t.) with 100 µl of PBS containing 5 µg α-GalCer, 50 µg of fd/α-GalCer bacteriophage conjugate, or with vehicle alone. Where indicated, 200 or 130 ng of α-GalCer was also used. After 24 h mice were sacrificed, spleens were harvested, and single-cell suspensions were prepared. Spleen cell suspensions were plated in U-bottomed 96-well plate at 2 × 10^5^ cells per well in RPMI medium supplemented with 10% FCS, 5 µM 2-ME, 1 mM glutamine, and 1 mM sodium pyruvate in the presence of indicated doses of free α-GalCer or with medium alone. For proliferation assays, 1 μCi of [3H] thymidine (PerkinElmer Life Sciences) was added to the wells after 60 h of culture, and cells were cultured for an additional 12 h. Cells were then harvested using a semi-automatic cell harvester FilterMate (PerkinElmer, CA, USA) and uptake of radioactivity was measured using the Top Count NTX microplate scintillation counter (PerkinElmer). Cell proliferation was expressed as a cpm fold increase vs unstimulated cells. IL-2 secretion was evaluated by ELISA using Mouse IL-2 ELISA MAX™ Standard, collecting cell culture supernatants (0.1 ml/well) after 60 h of culture.

### Analysis of iNKT and NK *In Vivo* Activation

Mice (*n* = 3/group) were injected i.v. with 100 µl of PBS containing 5 µg free or 50 µg of *fd* and fd/α-GalCer. Mice were sacrificed 3 h post-treatment. Splenic iNKT cells were analyzed for intracellular IFN-γ secretion by culturing 7.5 × 10^6^ spleen cells overnight in the presence of 10 µg/ml of the secretion inhibitor Brefeldin-A (BFA, Sigma-Aldrich). Cells were then harvested and IFN-γ production was evaluated by intracellular staining on gated CD3^+^NK1.1^+^ cells or CD3^−^NK1.1^+^ cells using APC-conjugated anti mouse CD3 monoclonal antibody (mAb) (17A2, Biolegend), PE-conjugated anti-mouse NK1.1 mAb (PK136, Biolegend), FITC-conjugated anti-mouse IFN-γ mAb (XMG1.2, Biolegend), and Leucoperm fixation and permeabilization kit (AbD Serotec, Oxford, UK). The IFN-γ release induced by 30 ng/ml of Phorbol 12-myristate 13-acetate (PMA, Sigma-Aldrich) plus 1 µg/ml of ionomycin (Sigma-Aldrich) was used as a positive control. Cells were analyzed using FACSCanto II flow-cytometer and DIVA software.

### *In Vivo* OVA_257–264_ T Cell Response Evaluation

Group of mice (*n* = 4) were primed (day 0) by subcutaneous injection with 100 µl of PBS containing fdOVA (SIINFEKL peptide) bacteriophages or fdOVA/α-GalCer bacteriophages (all delivering 1.5 µg of OVA_257–264_ peptide) and boosted (day 14) with the same amount of fdOVA bacteriophages delivering or not the α-GalCer. As control, mice were inoculated twice with vehicle alone (PBS).

At day 21, splenocytes were isolated and the frequency of OVA_257–264_ specific CD8^+^ T cells was assessed using FITC or APC conjugated anti-mouse CD8a mAb (53–6.7, Biolegend) and PE-H2Kb SIINFEKL MHC dextramers (Immudex) staining. Stained cells were analyzed by flow cytometry using a FACSCantoII. Results are expressed as the percentage of CD8^+^ gated cells that are positive for the MHC I/peptide dextramers.

IFN-γ-producing effector cells were evaluated by culturing 7.5 × 10^6^ spleen cells with OVA_257–264_ SIINFEKL synthetic peptide (10 μg/ml) for 5 h in the presence of BFA (Sigma-Aldrich). Cells were then harvested and IFN-γ production was evaluated by intracellular staining on gated CD8^+^ cells using FITC or APC conjugated anti-mouse CD8a mAb, PE conjugated anti-mouse IFN-γ mAb (XMG1.2 Biolegend), and Leucoperm fixation and permeabilization kit. The IFN-γ release induced by 30 ng/ml of PMA plus 1 µg/ml of ionomycin was used as a positive control. Data were acquired on FACSCanto II flow-cytometer and DIVA software.

### Therapeutic Vaccination Against B16 Tumor Cells

Naïve C57BL/6 mice were engrafted with 2 × 10^5^ B16 melanoma cells subcutaneously in the left flank. When tumors were palpable, mice were vaccinated with 100 µl volumes containing PBS, 2.5 µg α-GalCer, 50 µg of fdWT bacteriophages, or 50 µg of fd/α-GalCer. In the experiment with B16-OVA tumor cell line, mice were injected intratumorally twice, on day 0 and day 5, with 100 µl volumes containing PBS, 2.5 µg α-GalCer, 50 µg of fdOVA bacteriophages, 50 µg of fd/α-GalCer, or 50 µg of fdOVA/α-GalCer. Tumor growth was assessed three times weekly using caliper and recorded as tumor volume (mm^3^) according to the formula (*d*^2^ × *D*)/2, where *d* and *D* are the shortest and the longest diameters. Mice were culled once tumor size met or exceeded 1,500 mm^3^, in accordance with established guidelines. Survival was recorded as the percentage of surviving animals.

In another set of experiments, mice were sacrificed on day 12 and tumors and spleens were collected and homogenized. Tumors and spleen cells were filtered through a 70-µm cell strainer nylon mesh, and erythrocytes were lysed. Cells were then washed once with medium, resuspended in PBS + 5% FCS to a concentration of 1.5 × 10^6^ cells/ml and labeled with APC conjugated anti-mouse CD8a mAb and PE-H2Kb SIINFEKL MHC dextramers for flow cytometric analysis. 7-aminoactinomycin D (7-AAD) (BD) was used for live–dead cell discrimination.

### Statistical Analysis

Comparative analyses were performed using the analysis of variance (ANOVA) followed by a Dunnett’s multiple comparison test or Bonferroni *post hoc* comparisons. Analyses of survival were performed using the log-rank (Mantel–Cox) test. All the analyses were performed with the GraphPad Prism 5 program (GraphPad Software, CA, USA). In all cases, differences were considered statistically significant when *p* < 0.05.

## Results

### α-GalCer Conjugation to *fd* Filamentous Bacteriophage

**Figure 1:**
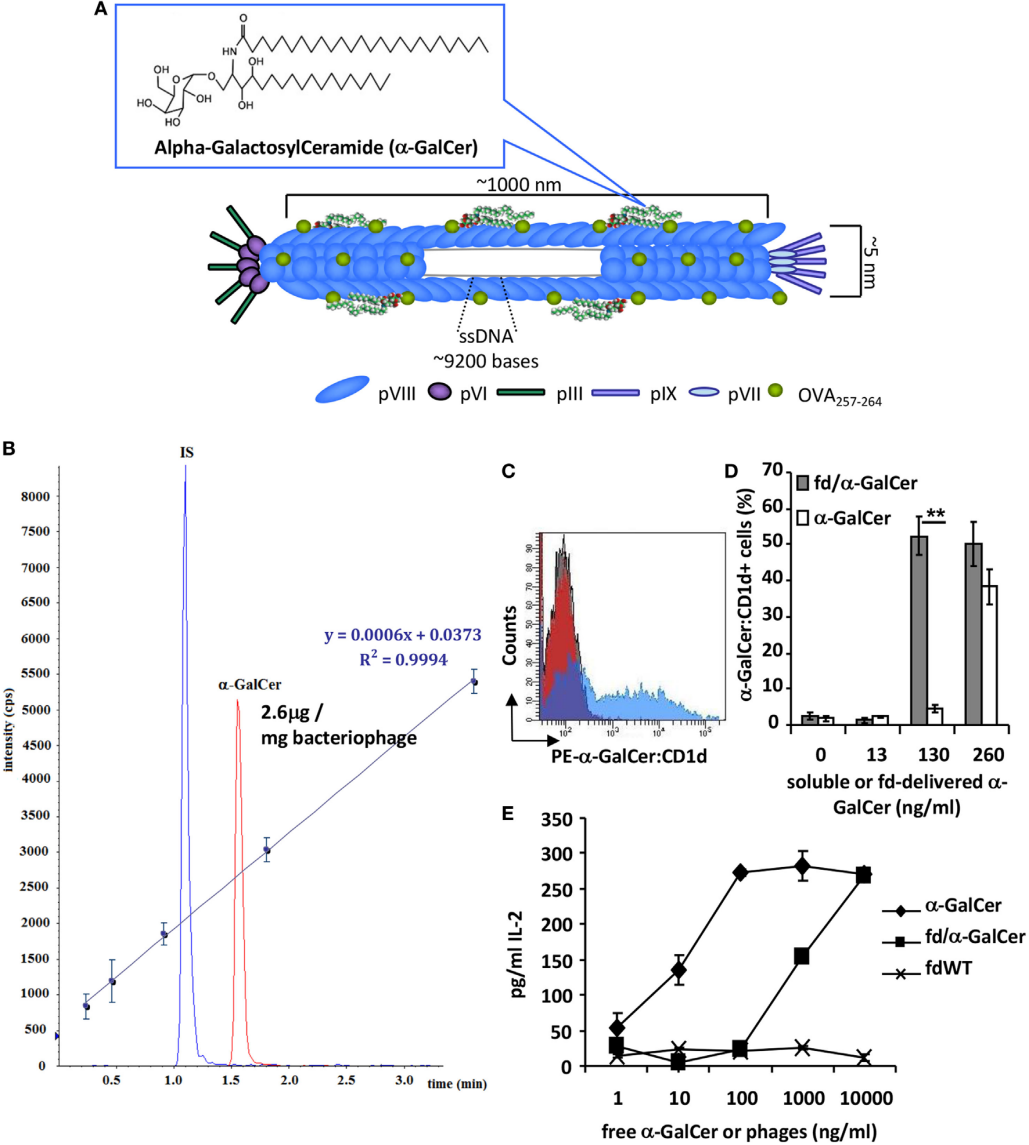
fd/alpha-GalactosylCeramide (α-GalCer) conjugate characterization. **(A)** Schematic image representing filamentous bacteriophage engineered for the expression of antigenic peptide and α-GalCer on the coat surface. **(B)** UPLC-multiple reaction monitoring profile of the organic extract of a solution containing 1.5 mg/ml of the bacteriophage conjugated with α-GalCer; IS, internal standard. The calibration curve obtained from five calibration points of α-GalCer STD (62.5, 125, 250, 500, and 1,000 ng/ml) spiked with fixed amount of IS (400 ng/ml) is reported along with equation parameters. **(C,D)** Dendritic cells (DCs) were cultured with free α-GalCer or fd/α-GalCer at different doses. After incubation, cells were stained with anti-α-GalCer:CD1d antibody and analyzed by flow cytometry. **(C)** Representative histogram overlay of DCs incubated with free α-GalCer or fd/α-GalCer at a dose of 130 ng/ml α-GalCer or fdWT. **(D)** Percentage of anti-α-GalCer:CD1d positive cells. Mean ± SD of two different experiments is reported. **p < 0.01 by unpaired two-tailed Student’s *t*-test. **(E)** The chart represents the IL-2 release of mouse Vα14 invariant natural killer T (iNKT) hybridoma cell line FF13 to α-GalCer delivered by phage particles. LPS-free filamentous bacteriophages were conjugated to α-GalCer, and presented by mouse DCs to stimulate iNKT hybridoma cells. Free form of α-GalCer was used as positive control. Supernatants were diluted 1:10 and assayed in duplicate. Mean ± SD is reported, one representative experiment of four is shown.

In order to exploit the ability of the major coat protein pVIII of the filamentous bacteriophage *fd* to bind hydrophobic lipids, α-GalCer was conjugated to LPS-purified bacteriophage particles (Figure 1A) in a 10:1 ratio (1.5 mg of bacteriophages: 150 µg α-GalCer), followed by ultracentrifugation on cesium chloride gradient to remove unbound lipids.

Alpha-GalactosylCeramide rapidly distributed on the pVIII protein thanks to hydrophobic interactions between the hydrophobic domains of the protein and the acyl chains of the lipid. The amount of α-GalCer conjugated to the bacteriophage vector was determined by quantitative mass analysis by a UPLC-MS/MS method. To this aim, the lipid was released from the conjugated vector by solvent extraction and the free glycosphingolipid was measured by multiple reaction monitoring (MRM) analysis. β-galactosylpalmitoylsphingosine was added as IS before extraction. The organic extract obtained as reported in the Section “Materials and Methods,” contained a measured absolute amount of α-GalCer of 0.196 µg/ml, indicating that 2.6% of the added galactosphingolipid was effectively loaded onto the phage particles (Figure 1B).

### fd/α-GalCer Ability to Activate iNKT Cells

We assessed the ability of α-GalCer loaded on phage particles to be presented by BMDCs and to activate iNKT cells *in vitro*. BMDCs were incubated with different doses of free α-GalCer or bacteriophage particles delivering α-GalCer. Cells were stained with a mAb recognizing the α-GalCer:CD1d complex and analyzed by flow cytometry. We found that fd/α-GalCer is able to be internalized by DCs and to efficiently induce α-GalCer presentation on CD1d molecule. In addition, at the dose of 130 ng only the α-GalCer carried by fd was displayed on CD1d molecule (Figures 1C,D).

Moreover, to investigate the efficiency of the antigen presentation of α-GalCer delivered by phage particles on CD1d, we co-cultured fd/α-GalCer pulsed BMDCs with the mouse Vα14 iNKT hybridoma cell line FF13. Bacteriophage-vectorized α-GalCer was presented by BMDCs, triggering activation of iNKT hybridoma, as assessed by IL-2 release (Figure 1E). According to mass analysis data, it is noteworthy that the highest used dose of bacteriophage (10,000 ng/ml) contains about 26 ng/ml of α-GalCer. The IL-2 production by hybridoma cells was due to the α-GalCer delivered on phage particles as demonstrated by the lack of capacity to induce IL-2 production from BMDCs pulsed with bacteriophage particles not-conjugated to the glycolipid (fdWT).

**Figure 2:**
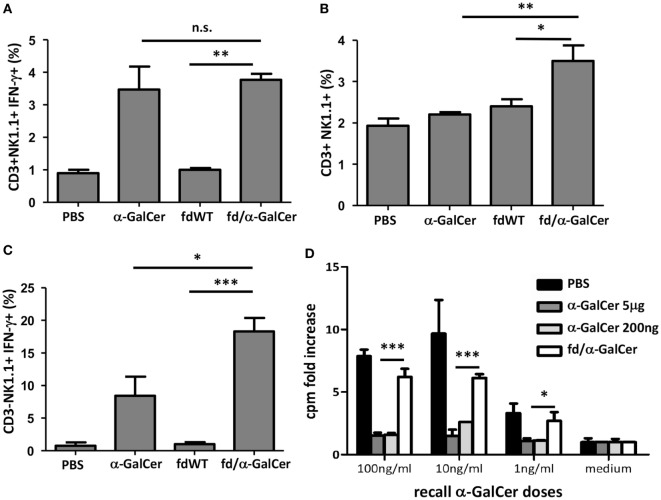
*In vitro* recall response of mice to intravenous alpha-GalactosylCeramide (α-GalCer) administration. Mice (*n* = 3/group) were injected intravenously with 50 µg of *fd* bacteriophages or 5 µg of free α-GalCer/mouse or vehicle (phosphate buffered saline, PBS). After 3 h, mice were sacrificed, spleen cells were collected and stained with the reported antibodies. **(A)** Percentage of INF-γ-producing CD3^+^NK1.1^+^ cells; average + SEM is reported. *p* < 0.001 by one-way analysis of variance (ANOVA) followed by a Dunnett’s multiple comparison test. **(B)** Percentage of CD3^+^NK1.1^+^ cells 3 h after intravenous injection of above-mentioned reagents. Average + SEM is reported. *p* < 0.01 by one-way ANOVA followed by a Dunnett’s multiple comparison test. **(C)** Percentage of INF-γ-producing CD3^−^NK1.1^+^. Average + SEM is reported. *p* < 0.001 by one-way ANOVA followed by a Dunnett’s multiple comparison test. *p < 0.05, **p < 0.01, ***p < 0.001. Abbreviation: ns, not significant. **(D)** *In vitro* recall response of mice to α-GalCer immunization. Mice (*n* = 3/group) were intravenously injected with 5 µg or 200 ng of free α-GalCer, 50 µg of fd/α-GalCer or vehicle alone (phosphate buffered saline, PBS). The day after, splenocytes were cultured with graded doses of α-GalCer or medium. After 3 days, proliferation was assessed by [3H] thymidine incorporation. Average + SEM is reported. *p* < 0.001 by two-way ANOVA and the Bonferroni multiple comparison test. *p < 0.05, **p < 0.01, ***p < 0.001.

We also tested the *in vivo* ability of bacteriophage/α-GalCer conjugates to activate a response, by injecting mice intravenously with 50 µg of fd/α-GalCer or 5 µg of free α-GalCer. We found that 3 h after the injection of fd/α-GalCer particles, CD3^+^NK1.1^+^ cells were activated, as measured by *ex vivo* analysis of IFN-γ production (Figure 2A). It is noteworthy that similar response was induced by the injection of the free α-GalCer and *fd*-conjugated α-GalCer even though the amount of lipid administered with the phage particles was less with respect to the free α-GalCer used. In fact, mass analysis demonstrated that approximately 130 ng of α-GalCer resulted bound to 50 µg of bacteriophages. Moreover, only the injection of vectorized glycosphingolipid was able to significantly increase the number of CD3^+^NK1.1^+^ in the spleen, compared to free α-GalCer injection (Figure 2B).

Importantly, notwithstanding the lower amount of lipid administered with bacteriophage particles, we found that fd/α-GalCer induced a higher percentage of NK cells able to produce IFN-γ compared to cells isolated from mice injected with free α-GalCer (Figure 2C).

### Recall Response to Intravenous α-GalCer Administration

It is known that free α-GalCer administered intravenously to mice, causes TCR down regulation on iNKT, and splenocytes from α-GalCer-injected mice loose their capacity to proliferate and produce cytokines upon *in vitro* re-stimulation with α-GalCer (13). In agreement with these reports we found that spleen iNKT cells isolated from mice injected with 5 µg of free α-GalCer were unable to proliferate when re-stimulated *in vitro* with the same lipid (Figure 2D). Since mass experiments showed that the amounts of α-GalCer bound to the phage particles is low, we also used a low dose of free α-GalCer in these experiments and similar unresponsiveness was observed (Figure 2D). In contrast, we observed that splenocytes from mice injected with vectorized α-GalCer, by conjugation to bacteriophages, were still responsive to α-GalCer re-stimulation *in vitro* in a dose-dependent manner (Figure 2D, empty bars).

### Antigen-Specific Immune Response Is Increased by the Adjuvant Effect of α-GalCer/Bacteriophage Conjugate

We then investigated the adjuvant effect of α-GalCer delivered by bacteriophages on the immunogenicity of a displayed antigenic determinant. For this purpose, α-GalCer was conjugated on the surface of *fd* particles displaying the ovalbumin (OVA) determinant SIINFEKL (OVA_257–264_).

**Figure 3:**
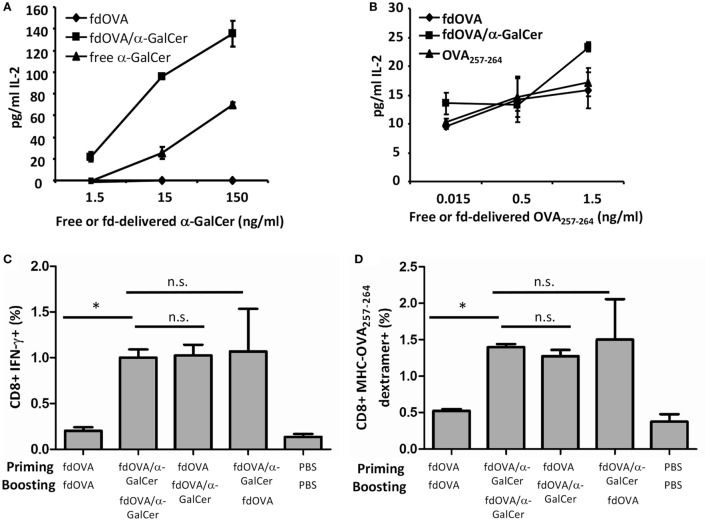
Adjuvant effect of vectorized delivery of alpha-GalactosylCeramide (α-GalCer) on bacteriophages in antigen-specific adaptive immune response. IL-2 release of mouse Vα14 invariant natural killer T hybridoma cell line FF13 **(A)** or OTI hybridoma cell line **(B)** in response to α-GalCer or OVA SIINFEKL peptide delivered by phage particles. LPS-free fdOVA filamentous bacteriophages were conjugated to α-GalCer, and fdOVA or fdOVA/α-GalCer were presented by mouse bone marrow-derived dendritic cells to stimulate FF13 or OTI hybridoma cells. Soluble form of α-GalCer was used as positive control in **(A)**. Synthetic OVA_257–264_ peptide was used as positive control in **(B)**. Supernatants were diluted 1:10 **(A)** or left undiluted **(B)** and assayed in duplicate. Mean ± SD is reported, one representative experiment of two is shown. **(C,D)** Group of mice (*n* = 4/group) were primed (day 0) and boosted (day 14) with fdOVA (SIINFEKL peptide) bacteriophages delivering or not the α-GalCer as indicated on the *x*-axis. As control, mice were inoculated twice with vehicle alone (phosphate buffered saline, PBS). At day 21, splenocytes were isolated and percentage of OVA_257–264_-specific CD8^+^ T cells producing IFN-γ **(C)** and percentage of H2Kb-SIINFEKL dextramer positive CD8^+^ T cells **(D)** were evaluated. Average + SEM is reported. Difference were statistical significant by one-way analysis of variance followed by a Dunnett’s multiple comparison test. *p < 0.05. Abbreviation: ns, not significant.

In Figures 3A,B we show that OVA antigen and α-GalCer co-delivered by phage particles are presented by DCs. BMDCs were incubated with fdOVA/α-GalCer conjugate, or with fdOVA alone. As controls, DCs were incubated with free α-GalCer or synthetic OVA_257–264_ peptide in the same amount as estimated on phage surface (see methods). BMDCs pulsed with fdOVA/α-GalCer are able to stimulate IL-2 production by either OTI hybridoma cells or iNKT hybridoma cells (Figures 3A,B). Of relevance, iNKT hybridoma cells produced higher amount of IL-2 when stimulated by DCs pulsed with fdOVAα–GalCer with respect to free α-GalCer.

C57BL/6 mice were immunized with bacteriophages expressing OVA_257–264_ peptide (fdOVA) or with fdOVA delivering α-GalCer (fdOVA/α-GalCer) and after 14 days, mice were boosted with the same bacteriophage particles, delivering or not α-GalCer. As control, mice were inoculated twice with only vehicle (PBS). On day 21, mice were culled and spleen cells were isolated and assayed for OVA_257–264_-specific cell response.

We found that immunization with fdOVA/α-GalCer particles developed an OVA_257–264_-specific IFN-γ-secreting T cell response. As illustrated in Figure 3, mice that received two fdOVA/α-GalCer administrations, showed increased OVA-specific CD8^+^ T cells producing IFN-γ compared to mice inoculated twice with fdOVA alone (Figures 3C,D).

Interestingly, the group of mice injected first with fdOVA and then with fdOVA/α-GalCer, showed similar results to two fdOVA/aGalCer injections, indicating that an adjuvant effect of α-GalCer administration *via* bacteriophage particles on the adaptive antigen-specific immune response can also be observed after a single administration of *fd* vectorized α-GalCer. As control, we treated a group of mice first with fdOVA/α-GalCer and then with fdOVA, obtaining comparable results to fdOVA/α-GalCer-treated group, suggesting that the fdOVA/α-GalCer administration either in priming or boosting is able to induce a higher CD8^+^ response.

### Therapeutic Vaccination With Bacteriophages in B16 Tumor-Bearing Mice

**Figure 4:**
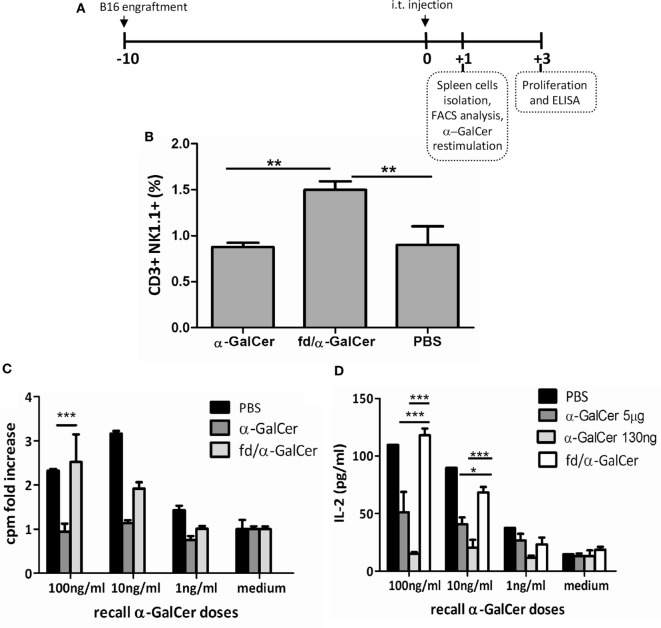
Invariant natural killer T response in mice injected intratumorally with fd-vectorized alpha-GalactosylCeramide (α-GalCer). Mice were inoculated with B16 melanoma cells and when tumor was palpable, mice were intratumorally injected with free α-GalCer, α-GalCer delivered by phage particles or vehicle alone, and sacrificed after 24 h. **(A)** Schematic representation of the experiment schedule. **(B)** CD3^+^NK1.1^+^ cells were evaluated in freshly isolated spleen of injected mice by FACS analysis and staining with anti-NK1.1 and anti-CD3 antibodies. Percentage of NK1.1^+^CD3^+^ positive cells ± SD is reported. *p* < 0.01 by one-way analysis of variance (ANOVA) followed by a Dunnett’s multiple comparison test. **p < 0.01. **(C,D)** Splenocytes were cultured with graded doses of α-GalCer. After 3 days, proliferation was assessed by [3H] thymidine incorporation **(C)**, and culture supernatants were evaluated for IL-2 release by ELISA **(D)**. Average + SEM is reported. *p* < 0.001 by two-way ANOVA and the Bonferroni multiple comparison test. *p < 0.05, ***p < 0.001.

We also tested the ability of bacteriophage particles coated with α-GalCer to mediate protection in a therapeutic anti-tumor vaccination setting. For this purpose, C57BL/6 mice were injected subcutaneously with B16 melanoma cells (day −10), and when tumors were palpable (day 0), mice were treated intratumorally with free α-GalCer, *fd* particles (fdWT), or fd/α-GalCer bacteriophages (Figure 4A). Although intratumoral route of administration is not applicable to all the type of cancer, it has been demonstrated that intratumoral administration of drugs offers several advantages over traditional routes of immunization, as reduced systemic toxicity due to lower diffusion in the body and high local concentration, which permits use of smaller amount of drugs (24).

We found that the intratumoral injection of α-GalCer vectorized on phage particles (fd/α-GalCer) was able to increase the number of iNKT cells, as demonstrated by FACS analysis on spleen cells 24 h later, in comparison with the number of iNKT cells observed in the spleens isolated from mice treated with free α-GalCer (Figure 4B). We also found that spleen cells derived from mice primed *in vivo* with fd/α-GalCer still retained the capability to proliferate and to produce cytokines like IL-2 when re-stimulated *in vitro* with increasing doses of free α-GalCer (Figures 4C,D). This response was not observed with cells isolated from mice injected *in vivo* with free α-GalCer, and also re-stimulated *in vitro* with free α-GalCer. Moreover, we used for i.t. injection a dose of free α-GalCer (130 ng), normalized according to the amount retained in the phage particles. Similar unresponsiveness to *in vitro* restimulation was observed (Figure 4D).

**Figure 5:**
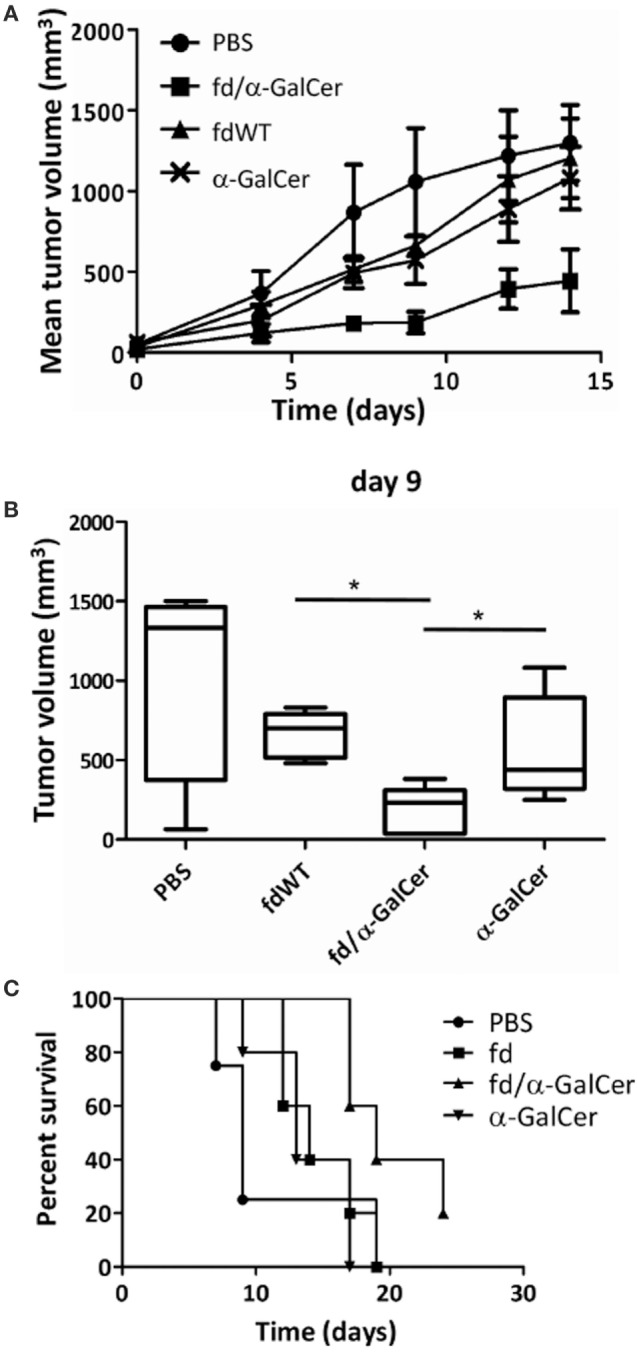
Therapeutic vaccination with filamentous bacteriophages fd/alpha-GalactosylCeramide (α-GalCer) is protective against a subcutaneous tumor challenge. C57BL/6 mice (*n* = 5/group) were engrafted with B16 melanoma cell line and when tumor was palpable, mice were injected two times subcutaneously into the tumor with phosphate buffered saline, free α-GalCer, fdWT, or fd/α-GalCer. **(A)** The chart shows the mean tumor size reached in each group. **(B)** Tumor volumes recorded at 9th day after vaccination are reported as box plot, showing the group median, quartiles, and extreme values. Median values are represented by the horizontal line. *p < 0.05 by one-way ANOVA followed by a Dunnett’s multiple comparison test. **(C)** Kaplan–Meier survival curves of B16-engrafted mice during the experiment. Differences among survival curves of α-GalCer-treated and fd/α-GalCer-treated mice are statistically different (*p* < 0.01) by log-rank (Mantel–Cox) test.

An important anti-tumor effect was also clearly assessed. fd/α-GalCer treatment resulted in a significant delay in the tumor growth, while administration of free α-GalCer or *fd* wild-type bacteriophages were less efficient in delaying tumor growth (Figures 5A,B). No significant differences were noted between groups of mice treated with fdWT or free α-GalCer. This protective effect was confirmed by the longer survival of mice receiving fd/α-GalCer (Figure 5C).

**Figure 6:**
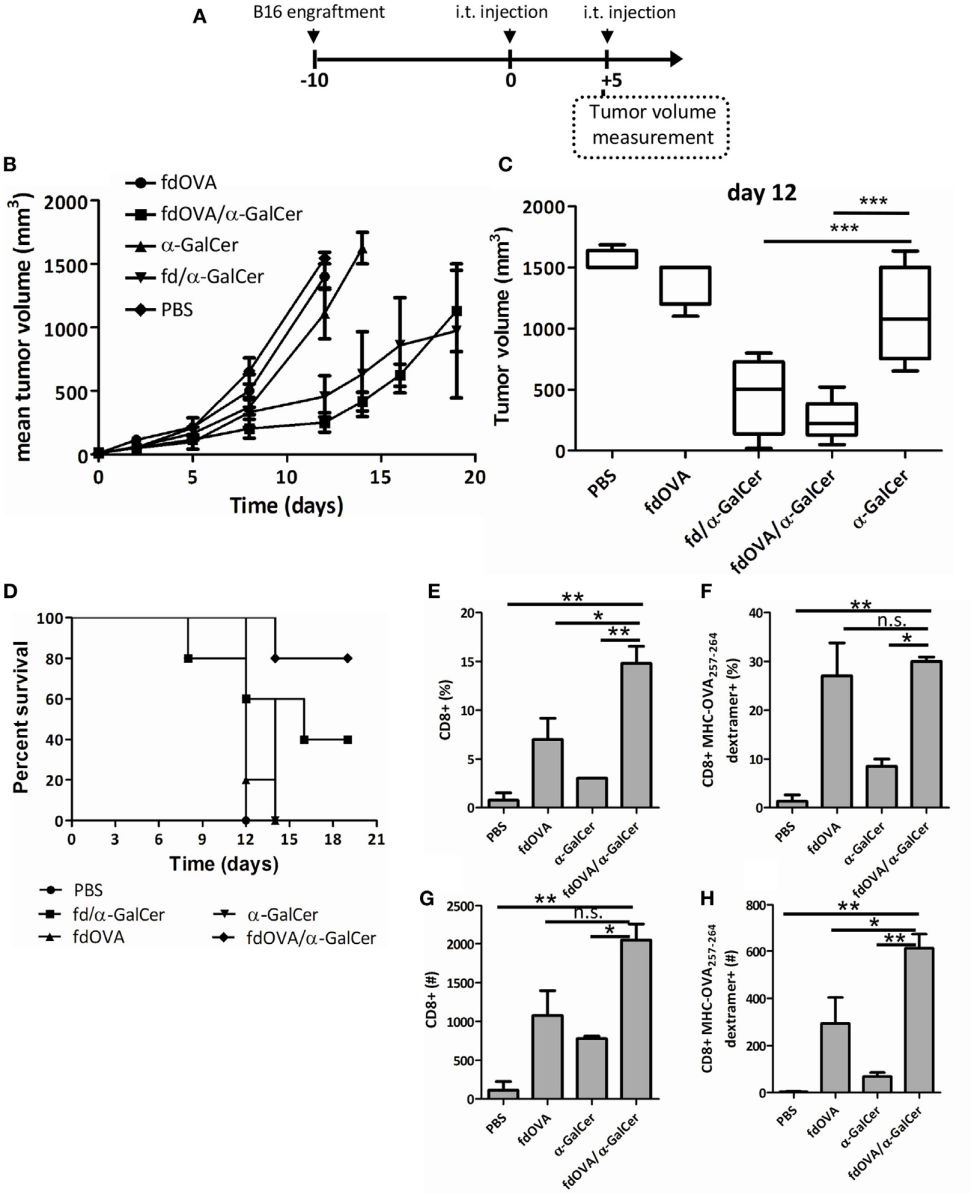
Filamentous bacteriophage *fd* co-delivering tumor antigen and alpha-GalactosylCeramide (α-GalCer) induces a stronger anti-tumor response. Mice (*n* = 5/group) were challenged with B16-OVA expressing melanoma cells. When tumor was palpable (day 0) mice were immunized intratumorally with fd/α-GalCer, fdOVA/α-GalCer, fdOVA, free α-GalCer, or with phosphate buffered saline. The injection was repeated at day 5, and tumor volume was monitored with a caliper over time. **(A)** Schematic representation of the experiment. **(B)** Tumor growth recorded during experiment. The chart shows the mean tumor size reached in each group. Line for each group ends once more than half of the group is culled. **(C)** Box plot of tumor volumes recorded at day 12 post vaccination. Median values are represented by the horizontal line. *p* < 0.001 by one-way analysis of variance (ANOVA) followed by a Dunnett’s multiple comparison test. ***p < 0.001 **(D)** Kaplan–Meier survival curves of B16-engrafted mice during the experiment. *p* < 0.05 between fdOVA/α-GalCer and free α-GalCer-treated mice group by log-rank (Mantel–Cox) test. **(E-H)** Groups of mice treated as above were sacrificed at day 12 post vaccination and tumor infiltrating lymphocytes were evaluated. Mean percentages **(E,F)** and absolute numbers **(G,H)** of CD8^+^ T cells gated on 7-AAD negative cells **(E,G)** and of H2Kb-SIINFEKL dextramer positive gated on CD8^+^ T cells **(F,H)** are reported. *p* < 0.01 by one-way ANOVA followed by a Dunnett’s multiple comparison test. *p < 0.05, **p < 0.01. Abbreviation: ns, not significant.

Finally, to investigate the capacity of fd/α-GalCer to induce a tumor-specific adaptive response in a vaccination model, mice were injected with hybrid bacteriophages co-expressing the OVA_257–264_ SIINFEKL peptide in the presence or absence of conjugated α-GalCer. C57BL/6 mice were engrafted (on day −10), with B16 melanoma cells engineered for the expression of ovalbumin protein. After tumor engraftment, mice received intratumorally fdOVA, fdOVA/α-GalCer, or fd/α-GalCer on day 0. The injection was repeated on day 5 and tumor growth was assessed over time (Figure 6A).

Priming and boosting mice with fdOVA/α-GalCer efficiently protected mice, as demonstrated by measuring tumor growth (Figures 6B,C). We observed a delay in tumor growth in mice immunized with fdOVA/α-GalCer phage particles, in comparison with mice treated with fdOVA or free α-GalCer.

Mice engrafted with B16-OVA cells after 2 injections with fdOVA/α-GalCer showed 80% survival rate at the end of the experiment, in comparison with 40% survival of mice treated twice with fd/α-GalCer in the absence of the specific OVA_257–264_ peptide (Figure 6D). In this latter group of mice, a variable response was observed, suggesting the presence of responder and not responder mice (Figure S1 in Supplementary Material). No increased survival was observed in mouse groups treated with two injections of free α-GalCer or fdOVA particles not delivering the galactosylceramide.

The analysis of tumor-infiltrating cells at day 12 from intratumor vaccination showed the presence of an increased percentage and absolute number of CD8^+^ T cells within the tumor bed in mice injected with fdOVA/α-GalCer compared to free α-GalCer and fdOVA-injected groups (Figures 6E,G), while the percentage of OVA_257–264_ specific T cells, as stained by SIINFEKL-MHC dextramer, was the same in fdOVA or fdOVA/α-GalCer-treated cells, both in tumors and in spleens (Figure 6F; Figure S2 in Supplementary Material). Importantly, the absolute number of tumor infiltrating OVA_257–264_-specific T cells was higher in mice treated with fdOVA/α-GalCer with respect to mice which received fdOVA (Figure 6H). These data indicate that fdOVA/α-GalCer bacteriophage particles are able to induce a general expansion of tumor-infiltrating T cells, probably due to the activity of α-GalCer, and then, an expansion of OVA_257–264_-specific cells, which are boosted by the phage particles co-displaying the OVA_257–264_ antigenic peptide.

## Discussion

Filamentous bacteriophage is a well characterized, powerful antigen delivery system that has been demonstrated to evoke long and sustained adaptive immune responses toward the antigens displayed on its surface (17–19). In addition, bacteriophage is able to activate also innate immune responses mainly *via* toll-like receptor pathways (23, 25).

To further improve the immune responses elicited by bacteriophage particles delivering antigenic peptides, in this work we exploited the capacity of the partially hydrophobic pVIII structural protein expressed on the phage surface to interact and bind the immunostimulating glycolipid α-GalCer. We found that the activation of iNKT *via* fd/α-GalCer, also rapidly induced transactivation of NK cells, which is considered to enhance the anti-tumor effect of α-GalCer treatment (26).

Furthermore, iNKT cells activated *in vivo* by fd/α-GalCer remained responsive to repeated stimulations, in contrast to long-term anergy observed upon free α-GalCer injection (13). The reason of iNKT unresponsiveness remains unclear, although it is known that injection of free α-GalCer rapidly induces the overexpression of inhibitory co-stimulatory programmed death (PD)-1 receptor (27). Nevertheless, blockage of PD-1/PD-L1 interaction does not restore full iNKT functions, indicating the presence of additional inhibitory mechanisms.

CD1d is expressed by many types of APCs which might all stimulate iNKT cells in principle. However, the APCs expressing appropriate levels of costimulatory molecules are those more efficient in inducing full activation of iNKT cells (13, 15, 28, 29). Studies involving conditional depletion of CD1d molecule on selected types of APC have demonstrated that different APCs have different capability to prime iNKT cells to glycolipid-specific responses.

In particular, when α-GalCer was presented by B cells a state of unresponsiveness was observed in iNKT cells (13, 30). In contrast, DCs resulted to be very active in stimulating iNKT cells with α-GalCer (31), and anergy could be further avoided by injection of α-GalCer-pulsed autologous DCs (15, 32). However, the dose of the injected lipid may be relevant in the induction of iNKT unresponsiveness by administration of α-GalCer-pulsed DCs (33). In addition, *ex vivo* manipulation of autologous human DCs appears to be a very expensive and time-consuming procedure.

For this reason, vectorizing α-GalCer on the surface of nanoparticles might be an interesting strategy to efficiently deliver the galactosylceramide to DCs in order to decrease the possibility of iNKT anergy. Indeed, nanoparticles are preferentially uptaken by DCs, and α-GalCer-coupled nanoparticles have been described to activate DCs without inducing anergy (34). The filamentous bacteriophage is composed by repeated and ordered subunits and for its particulate nature can be assimilated to a nature-made nanoparticle, and its use prevent unresponsiveness of iNKT.

Importantly, the simultaneous delivery of glycolipid and a MHC class-I-presented peptide to the same APC enhanced CTL cross priming as compared to separate administration of α-GalCer and protein antigen (35). We confirmed in our model that the co-delivery of tumor peptide and α-GalCer by phage particles increased the induction of antigen-specific CD8^+^ T cells.

The exact mechanism how α-GalCer facilitates priming and expansion of OVA-specific CD8^+^ T cells remain to be investigated. We envisage two, non-alternative mechanisms. The first is due to the recruitment of iNKT cells next to DCs that internalized fdOVA/α-GalCer. Recruited iNKT cells may immediately induce maturation of DCs and also the optimization of their Ag-presentation and priming capacities of CD8^+^ cells. These effects might be facilitated also by the large numbers of iNKT cells and their immediate response (within a few minutes) to α-GalCer stimulation. The activation of iNKT cells might explain the reports in which it was found superior to those elicited by TLR agonists in inducing specific anti-tumor CD8^+^ T cell responses (36).

The second mechanism may be ascribed to the fact that bacteriophage, after internalization localizes into the endocytic cellular compartments (23), where loading of glycolipids on CD1d molecule also occurs.

Overall, our findings suggest that filamentous bacteriophage is a powerful tool to deliver immunogenic lipid molecules together with antigenic peptides.

Growing interest is focused on the role of lipids as key stimulatory molecules of the immune response. Either naturally derived non-self lipids, microbial lipids (i.e., peptidoglycan, lipoteichoic acid, and lipopolysaccharides) or self lipids (i.e., phospholipids, sphingomyelin, and oxidized lipoproteins) are known to trigger the immune system (37–41), and co-administration of stimulatory lipid molecules and antigens of interest in vaccination seems a promising manner to formulate novel vaccines including anti-tumor vaccines.

The use of filamentous bacteriophages complexed with lipid molecules might represent an efficient, fast, and cheap way to generate new vaccines capable of efficient priming and expansion of T cells and also devoid of chemical adjuvants. Our approach can be further improved by developing phage-based antigen delivery systems loaded with α-GalCer and simultaneously expressing antigens target of the vaccine and molecules delivering the phage to specific cell subsets. Bacteriophages are generally considered safe (42, 43) and cheap to prepare and purify (44, 45), thereby they might represent new tools for the next generation of vaccines.

## Ethics Statement

This study was carried out in accordance with European Union Laws and guidelines (European Directive 2010/63/EU). The study was approved by our institutional review board and the animal procedures (i.e., immunization, sacrifice) were performed according to rules approved by the ethics committee (permission no. 79/2014-PR).

## Author Contributions

Conceptualization: RS and PB. Methodology: RS, AC, and PB. Investigation: RS, LA, PB, DC, LG, and AC. Writing: RS and PB. Review and editing: LA and AC. Funding acquisition: PB. Supervision: PB. All authors read and approved the final version of the manuscript.

## Conflict of Interest Statement

RS and PB are inventors of the patent no. PCT/IB2018/050525 “Phage conjugates and uses thereof.”
