# Supplementary material for: Vectorized Delivery of Alpha-GalactosylCeramide and Tumor Antigen on Filamentous Bacteriophage fd Induces Protective Immunity by Enhancing Tumor-Specific T Cell Response
Source: Front Immunol. 2018 Jun 28;9:1496. doi: 10.3389/fimmu.2018.01496 (PMC6031736; doi:10.3389/fimmu.2018.01496)
Supplement: Supplementary file 1 [file Presentation_1.ppt]

## Slide 1
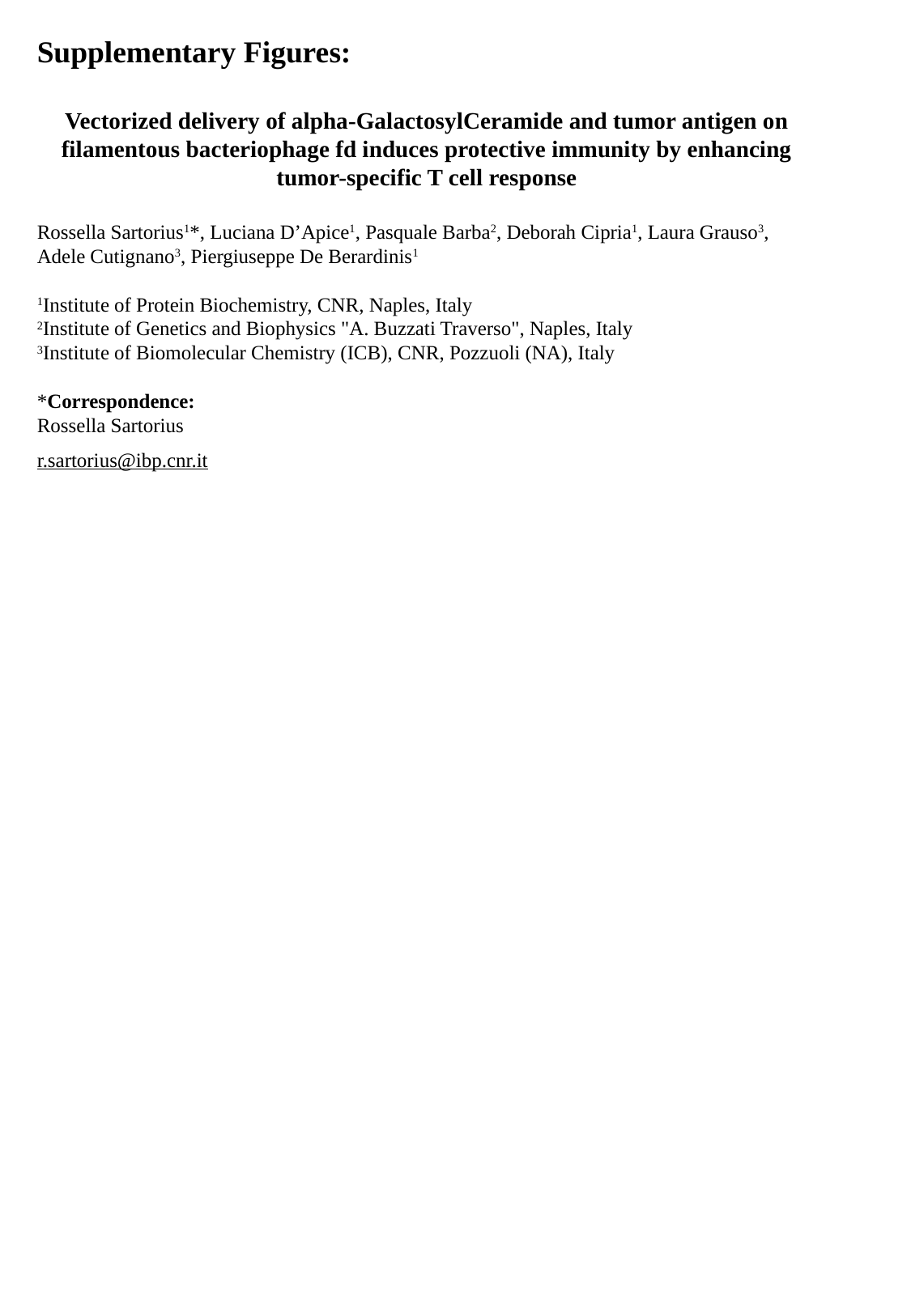

Supplementary Figures:
Vectorized delivery of alpha-GalactosylCeramide and tumor antigen on filamentous bacteriophage fd induces protective immunity by enhancing tumor-specific T cell response
Rossella Sartorius1*, Luciana D’Apice1, Pasquale Barba2, Deborah Cipria1, Laura Grauso3, Adele Cutignano3, Piergiuseppe De Berardinis1
1Institute of Protein Biochemistry, CNR, Naples, Italy
2Institute of Genetics and Biophysics "A. Buzzati Traverso", Naples, Italy
3Institute of Biomolecular Chemistry (ICB), CNR, Pozzuoli (NA), Italy
*Correspondence:
Rossella Sartorius
r.sartorius@ibp.cnr.it

## Slide 2
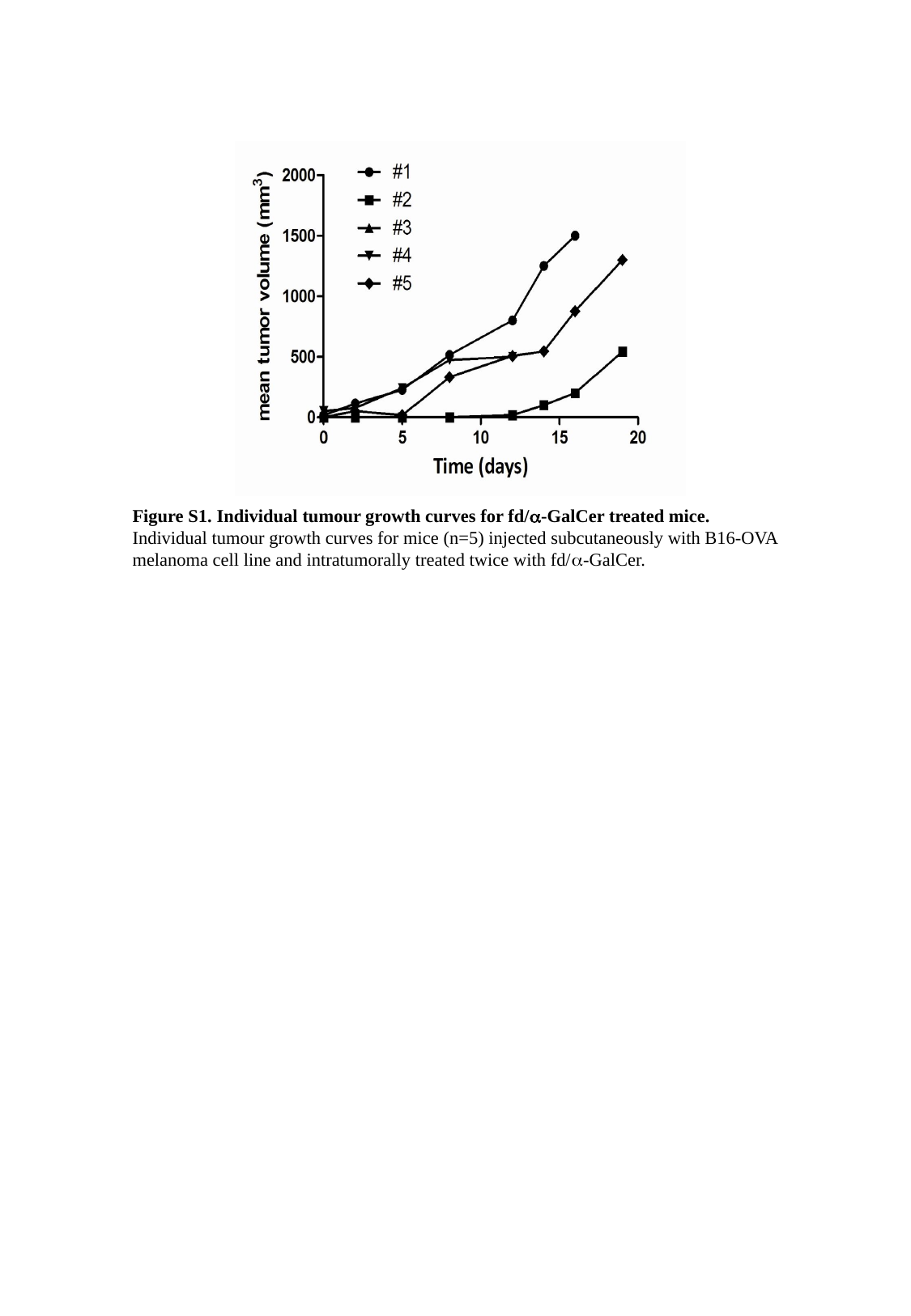

# Figure S1. Individual tumour growth curves for fd/α-GalCer treated mice.
Individual tumour growth curves for mice (n=5) injected subcutaneously with B16-OVA melanoma cell line and intratumorally treated twice with fd/α-GalCer.

## Slide 3
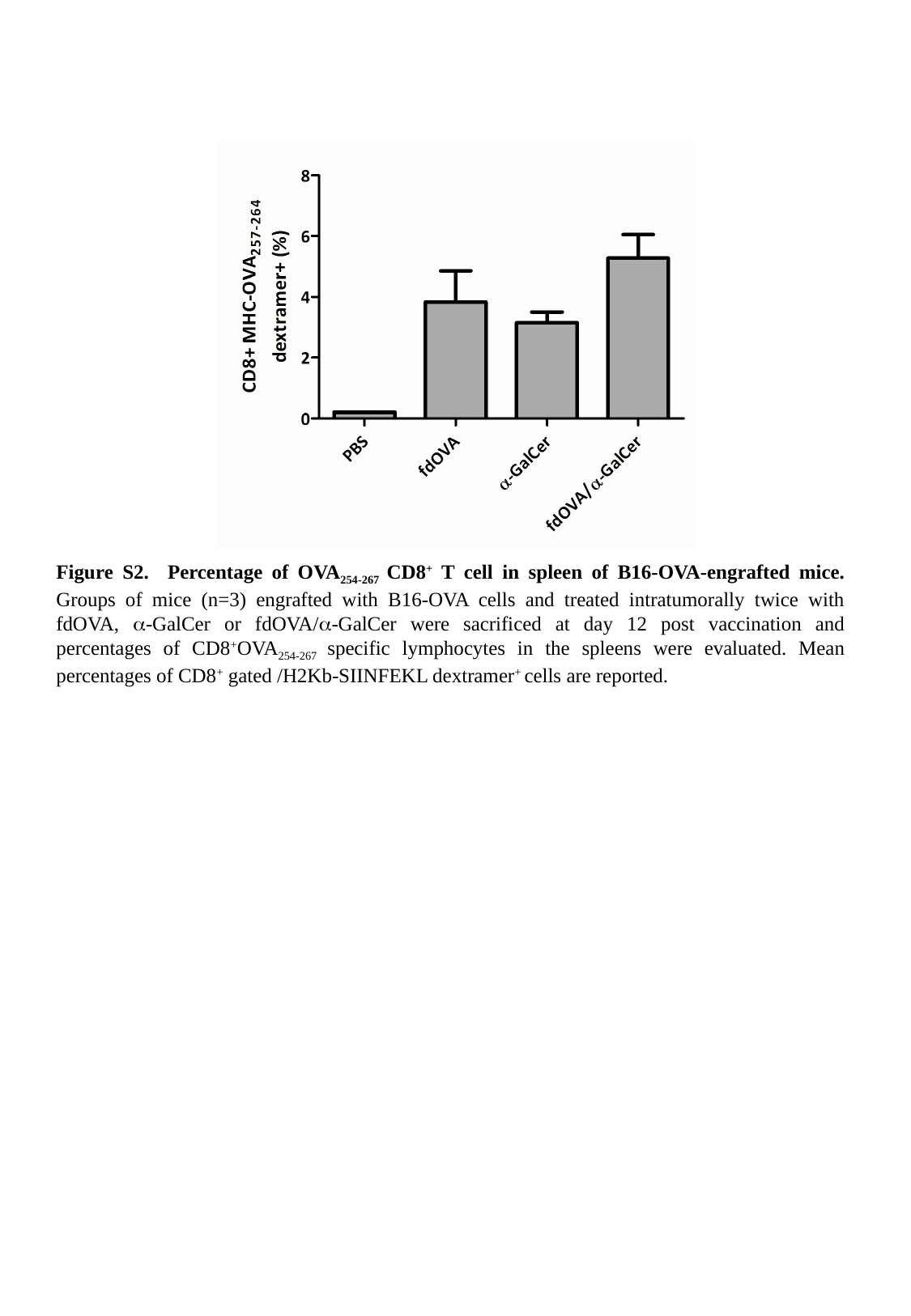

Figure S2. Percentage of OVA254-267 CD8+ T cell in spleen of B16-OVA-engrafted mice. Groups of mice (n=3) engrafted with B16-OVA cells and treated intratumorally twice with fdOVA, α-GalCer or fdOVA/α-GalCer were sacrificed at day 12 post vaccination and percentages of CD8+OVA254-267 specific lymphocytes in the spleens were evaluated. Mean percentages of CD8+ gated /H2Kb-SIINFEKL dextramer+ cells are reported.
